# Fetal Cerebral Artery Mitochondrion as Target of Prenatal Alcohol Exposure

**DOI:** 10.3390/ijerph16091586

**Published:** 2019-05-07

**Authors:** Anna N. Bukiya

**Affiliations:** Department Pharmacology, College of Medicine, The University of Tennessee Health Science Center, Memphis, TN 38163, USA; abukiya@uthsc.edu; Tel.: +1-901-448-2128

**Keywords:** maternal drinking, intrauterine alcohol, alcohol in utero, nonhuman primate, basilar artery, neurovascular unit

## Abstract

Prenatal alcohol exposure results in an array of developmental abnormalities known as fetal alcohol spectrum disorders (FASDs). Despite the high prevalence of FASDs, therapeutic interventions against accidental or intended exposure of developing fetuses to alcohol are limited. This review outlines current knowledge about mitochondria in cerebral blood vessels as a potential target for anti-FASDs intervention. First, it describes the multifaceted role of mitochondria in maintaining the cerebral artery diameter as shown in adult tissue. Second, current literature on alcohol-driven damage of mitochondrial morphology and function in several fetal tissues, including liver, heart, and brain is summarized. The functional consequences of alcohol exposure in these organs include morphological enlargement of mitochondria, increased oxidative stress, and alteration of cellular respiration. These studies point to a tissue-specific effect of alcohol on mitochondrial function and a particular vulnerability of fetal mitochondria to alcohol exposure when compared to adult counterparts. Third, recent work from our group describing persistent changes in fetal baboon cerebral artery proteome following three episodes of prenatal alcohol exposure is reviewed. In conclusion, the consequences of prenatal alcohol exposure on cerebral artery mitochondria constitute an open field of investigation and, eventually, a point of therapeutic intervention against FASDs.

## 1. Introduction

Alcohol (ethyl alcohol, ethanol) is one of the most widely consumed psychoactive substances in the world [1]. Despite major research and education efforts describing the deleterious effects of excessive alcohol consumption on human health, alcohol continues to be consumed by pregnant women in both industrialized and developing countries [2,3]. As documented by numerous studies, maternal alcohol consumption with sharp and high peaks of maternal blood alcohol levels (BAL) above 80 mg/dL poses the highest risk of fetal developmental abnormalities [4,5,6]. These abnormalities encompass multiple aspects of fetal development, ranging from subtle cognitive delay to severe morphological and neuronal anomalies. The entire spectrum of developmental changes triggered by maternal alcohol consumption is termed fetal alcohol spectrum disorders (FASDs) [7,8]. The most severe cases of prenatal alcohol damage are manifested as fetal alcohol syndrome (FAS) [7]. Although there are different diagnostic trees for severe prenatal damage by alcohol, all converge on the most distinctive facial characteristics (short palpebral fissures, a smooth philtrum, a thin upper vermillion border) and growth deficiency as pre- or postnatal weight/height at or below the 10th percentile. [7]. A wide range of behavioral and cognitive abnormalities that result from prenatal alcohol exposure is often specific toward deficits in magnitude comparisons and in eyeblink conditioning [7]. The global prevalence of FASDs among children and youth is estimated at 0.77% [2,9,10,11,12]. However, FASDs prevalence is as high as 30% in particular geographic areas with high occurrence of maternal binge alcohol consumption (such as South Africa) [10].

Currently, there is no readily available cure for intended or accidental fetal exposure to alcohol [13,14]. One of the reasons is the limited mechanistic understanding of FASDs pathophysiology. This limitation arises largely from the peculiar pharmacological profile of the ethanol molecule, which allows it to readily cross cell membranes and freely distribute among body compartments. Thus, ethanol targets multiple proteins, cellular processes, organs, and systems [15,16,17]. The fetal brain is the most severely affected organ, exhibiting both structural and functional abnormalities in response to maternal alcohol consumption [18,19]. Recently, the effects of alcohol on fetal cerebrovascular function started receiving increasing attention, as brain energetic demands are usually met by a constantly adapting blood supply [20]. Such an adaptation is initiated at the level of cerebral arteries and spans to microvessels that penetrate the brain parenchyma and engage in the formation of neurovascular units [21,22].

Within the cells, mitochondria constantly power adaptation to dynamic changes in metabolic demands. Thus, it comes as no surprise that vascular mitochondria dysfunction is implicated in the progressive decline observed during aging, development of neurocognitive disorders (such as Alzheimer’s disease), and substance abuse [23,24,25,26,27]. The deleterious effects of alcohol exposure on mitochondrial morphology and function within fetal tissues have been widely documented [28]. Moreover, mitochondrial dysfunction in fetal cardiomyocytes has been proposed as one of the driving forces behind the development of heart pathology present in severe cases of fetal damage by alcohol exposure in utero such as in FAS [29]. The majority of studies, however, focus on fetal neurons, heart, and liver, which leaves many unanswered questions on the role of cerebrovascular mitochondria in the sequelea triggered by prenatal alcohol exposure. This review summarizes findings that help to fill this gap in knowledge.

First, a multifaceted role of mitochondria in maintaining cerebral artery diameter as shown in adult tissue is described. Second, current literature on alcohol-driven damage of mitochondrial morphology and function in several fetal liver, heart, and brain is presented. Third, recent work from our group describing persistent changes in fetal baboon cerebral artery proteome following three episodes of prenatal alcohol exposure is discussed. The review concludes with the prospective view on the future of cerebrovascular mitochondria research in the field of FASD pathology and therapeutic developments. 

## 2. Mitochondria: Basic Morphology and Function

**Figure 1:**
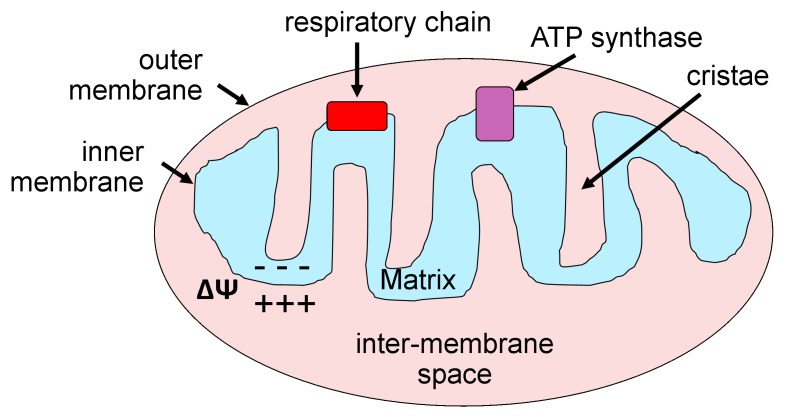
Principal structural and functional components of cellular mitochondrion. ATP: Adenosine triphosphate.

Mitochondria are formed by two membranes, termed outer and inner. The inner has multiple extensions called “cristae” with the intracristae space being referred to as the matrix (Figure 1). Mitochondrial morphological components serve their unique functions. For instance, the Krebs cycle occurs in the matrix, while protein complexes associated with the electron transport chain are embedded into the inner membrane. Mitochondrion has its own DNA and protein-synthesizing machinery. However, approximately one-half of mitochondrial proteome is encoded by nuclear DNA, which is transported to mitochondria. Mitochondrion morphology and basic functioning are extensively reviewed elsewhere [26,30,31]. The size and shape of mitochondria are determined by fusion and fission [31,32,33]. Moreover, modulation of these basic processes by cell signaling results in modifications of mitochondria shape and function [33,34]. Thus, elongation of mitochondria triggers senescence-like state in cellular cultures, which is accompanied by a decrease in mitochondrial membrane potential and increased generation of ROS [35]. Mutations in genes that encode proteins affecting fusion and fission are associated with human disease [36,37,38,39]. 

A basic mitochondrial feature is the maintenance of membrane potential across the inner membrane. This membrane potential is usually set around −180 mV and provides a driving force for electron transport. A plethora of potassium channels located within the mitochondrial inner membrane has been implicated in mitochondrial membrane potential maintenance. These channels include voltage- and Ca^2+^-gated potassium channels of large conductance (BK_Ca_), potassium channels of small (SK) and intermediate (IK) conductance, as well as adenosine triphosphate (ATP)-sensitive potassium channels (K_ATP_) [40,41]. The latter are composed of inwardly rectifying K^+^ 6.1 channel and sulfonylurea receptor SUR-2 subunits and have been extensively used as pharmacological targets to evoke mitochondrial depolarization, which could be accompanied by reactive oxygen species (ROS) production [42,43]. 

Mitochondrion has been conventionally considered as a main energy producing cellular organelle, in which the highly conserved process of oxidative phosphorylation occurs [26,31,44]. However, in recent years, a growing number of studies have documented mitochondria function as a source of signaling molecules which thus plays major roles in a plethora of physiological and pathological processes [26,43]. Indeed, at the cellular level, mitochondria are central not only to maintenance of cellular ATP and redox potential but also for modulating two intracellular messengers: ROS and Ca^2+^ itself [45,46,47,48,49,50,51]. Mitochondria capture Ca^2+^ from intracellular space via a low-affinity inner membrane mitochondria Ca^2+^ uniporter (MCU), while Ca^2+^ release may occur via several mechanisms, such as the mitochondrial permeability transition pore (PTP) and the Na^+^/Ca^2+^ exchanger [52,53].

Mitochondria compartmentalize glutathione (GSH), which is the main nonprotein thiol in cells where functions are dependent on the redox-active thiol of GSH cysteine moiety. The latter serves as a cofactor for an array of antioxidant and detoxifying enzymes [54,55,56]. Mitochondrial GSH levels are often used as a reader of mitochondrial ROS detoxification, with low GSH leading to oxidative stress and accompanying prevalent health disorders [54,55,56].

Mitochondria also harbor a fraction of alcohol dehydrogenase (ADH) and aldehyde dehydrogenase (ALDH) enzymes, which provide the first and the second steps in the major oxidative pathway of alcohol metabolism [57,58,59,60]. Both ADH and ALDH use the cofactor nicotinamide adenine dinucleotide (NAD^+^), which is reduced to NADH. Alcohol consumption results in a reduced NADH/NAD^+^ ratio, shifting cellular redox balance [61]. 

## 3. Cerebrovascular Mitochondria

Within cerebral arteries, mitochondria control a plethora of cellular processes, including apoptosis [62,63], maintenance of arterial tone, constrictive or dilatory responses to physiological/pharmacological stimuli, adaptation to environmental insult [27,30,43,64], and vascular aging [65,66]. Indeed, mitochondrial size is increased with age, as reported for vascular smooth muscle of 3 versus 18 months-old Sprague–Dawley rats [67]. A subset of aged mitochondria is apparently elongated with length exceeding width dimensions over 3-fold. Aged mitochondria are also less mobile when compared to young tissue, which exhibits mitochondrial motions that reach 12 μm within 10 min [67]. Notably, mitochondrial motility detected by high-speed fluorescence imaging is characteristic of a proliferative state, as shown in vascular smooth muscle of resistance-size cerebral arteries of guinea pig [68]. Conceivably, restriction of mitochondrial fission prevents vascular smooth muscle proliferation at single cell and resistance size artery levels [68]. Thus, it has been proposed that mitochondrial motion might be critical for cell proliferation and recovery from vascular injury [67,68]. 

A study on 3- versus 24-months-old mice demonstrated increased ROS production in cerebral arteries of the latter group in response to in vitro pressurization at 140 mmHg [66]. Intracellular vascular mechanisms that link cerebrovascular mechanosensitivity to mitochondrial ROS level remain elusive. Another link between fundamental arterial properties and mitochondrial function is presented by nitric oxide: Application of nitric oxide synthase inhibitor to cerebral arteries from adult rats results in increased mitochondrial respiration [69]. Importantly, there are gender differences in cerebral artery mitochondrial characteristics. For example, cerebral arteries from adult (8- to 10-weeks-old) female rats are larger in mass and also characterized by elevated ATP production, proton leak, maximal respiration, and spare respiratory capacity [43,69]. Moreover, application of K_ATP_ channel activator diazoxide to female rat cerebral arteries results in decreased mitochondrial respiration, while having little effect on respiration in arteries from male rat donors [69].

Several pathological conditions affecting vascular function are also associated with alterations of mitochondria characteristics. Maximal respiration is increased in cerebral microvessels of Zucker obese insulin-resistant rats when compared to the lean phenotype [70]. Although mitochondria respiration and protein levels remain unaltered at the point of insulin resistance, cerebral arteries from insulin-resistant Zucker obese rats exhibit reduced vasodilation in response to K_ATP_ channel activator diazoxide [71]. At early stages of type 2 diabetes development, however, basal mitochondrial respiration and proton leak are significantly decreased in the large cerebral arteries from Zucker diabetic fatty obese rats when compared to their age-matched (14-weeks-old) lean counterparts [72]. In the same experimental setting, superoxide production is increased in Zucker diabetic fatty obese rats, and this increase cannot be counterbalanced by the exogenous superoxide dismutase [72]. These findings point to the dynamic nature of mitochondrial sensing of pathology.

In a cerebrovascular ischemia/reperfusion model, mitochondria retain functional state for up to at least 48 h following transient occlusion of middle cerebral artery (MCA) in rats [73]. This finding prompted speculation that mitochondria could be considered as a therapeutic target during ischemia/reperfusion [73]. However, in male rats, oxygen consumption rate is increased in ipsilateral (ischemic) hemisphere arteries when compared to contralateral (non-ischemic) counterparts in the same transient MCA occlusion model [74]. Mitochondria depolarization also significantly increases Ca^2+^ sparks in ipsilateral but not contralateral cerebral arteries [74]. Although many studies on mitochondria role in vasculature are ongoing, available data leave no doubt about the critical role of mitochondrial function in vascular physiology and pathology. 

## 4. Mitochondrial Function and Control of Cerebral Artery Diameter

At the level of cellular function, mitochondrion regulates cerebrovascular myocyte Ca^2+^ signaling. In particular, mitochondrial depolarization within rat posterior cerebral and cerebellar artery myocyte results in decreased Ca^2+^ sparks and waves yet elevates intracellular global Ca^2+^ [75]. The reduction in Ca^2+^ spark frequency and amplitude leads to a reduction in outward K_Ca_ currents; this reduction is further deepened by reduced efficiency of spark-K_Ca_ current coupling [75]. Mitochondria depolarization-driven decrease in K_Ca_ is attenuated by permeability transition pore block. Thus, it has been proposed that mitochondrial depolarization opens mitochondrial PTP in the inner membrane leading to inhibition of Ca^2+^ sparks and transient K_Ca_ currents [75]. These findings contrast with other reports pointing to vasodilatory properties of mitochondrial depolarization (see below). The discrepancy may be explained by the differential time-course and pharmacological profile of the studies. 

**Figure 2:**
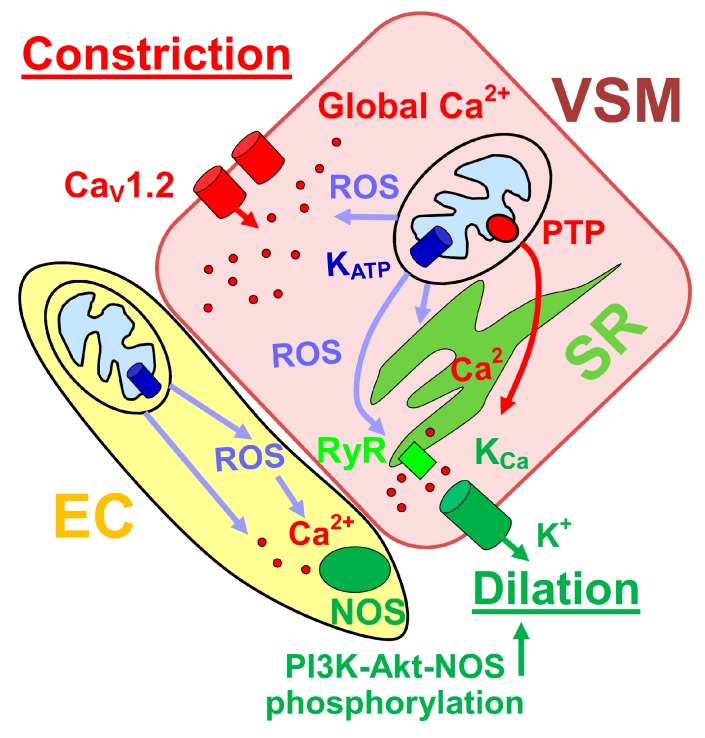
Pathways of mitochondria-mediated control of cerebral artery diameter. A balance between constriction and dilation is achieved by a plethora of mechanisms. Vasoconstrictive mechanisms include vascular myocyte mitochondrial amplification of hydrogen peroxide signaling that result in activation of Ca^2+^ entry into the myocyte and upregulation of Ca_V_1.2 channel transcription. Increase in vascular smooth muscle (VSM) global Ca^2+^ is accompanied by an opening of the permeability transition pore (PTP) and a decreased Ca^2+^ spark and K_Ca_ [75]. Dilatory mechanisms include pathways within VSM as well as endothelial cells (EC). Mitochondrial depolarization via K_ATP_ channel activation within VSM results in ROS-dependent, and independent activation of Ca^2+^ sparks release from sarcoplasmic reticulum (SR). Ca^2+^ sparks activate voltage- and Ca^2+^-gated potassium channels of large conductance (K_Ca_). The latter generate outward potassium currents that negatively feedback on depolarization-driven Ca^2+^ entry into the myocyte [78]. VSM-mediated vasodilation is further bolstered by ROS-dependent and independent mechanisms that trigger phosphoinositide 3-kinase (PI3K)–Akt–NOS phosphorylation within EC and thus increase production of vasodilator nitric oxide. Mitochondrial depolarization within EC is accompanied by an increase in EC intracellular Ca^2+^.

Mitochondria depolarization-driven decrease in transient K_Ca_ currents is still observed in the presence of voltage-gated calcium channel blocker diltiazem [75]. This is noteworthy as mitochondrion location correlates with the spatial distribution of L-type Ca^2+^-channels as shown in rat basilar and cerebral artery myocytes [76]. Moreover, vascular myocyte mitochondrial amplification of hydrogen peroxide signaling activates Ca^2+^ entry in the form of Ca^2+^ sparklets into the myocyte [76] and upregulates Ca_V_1.2 channel transcription (Figure 2) [77]. The latter effect is mediated by NF-kB activation by mitochondrial ROS that are produced in response to IP3 receptor-mediated release of Ca^2+^ from sarcoplasmic reticulum [77]. At the whole organ level, artery treatment with mitochondria-targeted antioxidant mitoTEMPO decreases production of hydrogen peroxide in presence of angiotensin II (AngII) and also diminishes AngII-induced vasoconstriction. In vivo treatment of Sprague–Dawley rats with mitoTEMPO also diminishes pressure-induced vasoconstriction in presence of nitric oxide production blocker L-NAME [76]. 

Despite the involvement of mitochondria in vasoconstrictive mechanisms, a plethora of mitochondria-mediated vasodilatory responses has also been reported in the cerebral vasculature. It has been shown that mitochondria-derived ROS give rise to an increase in transient K_Ca_ currents that are triggered by mitochondrial ROS-induced activation of Ca^2+^ sparks and provide negative feedback on depolarization-induced Ca^2+^ entry [79]. However, another study points to the ROS-independent mechanism of mitochondrial depolarization-mediated dilation of rat cerebral arteries. In particular, Katakam et al. [80] compared vasodilatory responses in the presence of mitochondrial K_ATP_ channel activator diazoxide that activates ROS production (likely via inhibition of succinate dehydrogenase [81]) and ROS-free activator of K_ATP_ channels BMS-191095 (BMS). Both agents produce vasodilation in de-endothelialized arteries, yet ROS scavenging does not affect vasodilation triggered by BMS [80]. Both diazoxide and BMS ultimately converge on increased Ca^2+^ spark generation and activation of BK_Ca_ channels to oppose constrictive machinery of cerebral vessels [80]. While ROS-dependent increase in Ca^2+^ spark generation may be mediated via redox modulation of Ca^2+^ spark-generating ryanodine receptors (RyRs) within sarcoplasmic reticulum (SR), the ROS-independent pathway may involve electrical coupling of mitochondria with SR to allow sensing of mitochondrial depolarization (Figure 2) [80].

Mitochondrion control over cerebral artery diameter is also exerted at the endothelial layer, as diazoxide and BMS-induced dilation of cerebral arteries is larger in vessels with intact endothelium when compared to de-endothelialized cerebral arteries [82]. Mitochondrial depolarization-induced vasodilation in arteries with intact endothelium is diminished by inhibition of nitric oxide synthase (NOS) or phophoinositide-3 kinase (PI3K). The latter is critical for diazoxide-driven phosphorylation of Akt and NOS by diazoxide and BMS. As in smooth muscle, scavenging of ROS in arteries with intact endothelium reduced the vasodilation caused by diazoxide but not that from ROS-free BMS treatment [82]. Notably, both diazoxide and BMS increased intracellular Ca^2+^ in cultured rat brain microvascular endothelial cells [82]. Thus, a rather complex picture emerges of ROS-dependent and independent mechanisms that bolster mitochondrial depolarization-driven dilation of cerebral arteries with intact endothelium (Figure 2). Of note, cerebral artery endothelial mitochondrion content is higher than in other vascular beds. This mitochondrial enrichment of cerebrovascular endothelium has been suggested to arise from high energetic demands of the blood–brain barrier [83]. 

A critical feature for the proper function of cerebral arteries is their ability to develop and maintain constant diameter independent of changes in systemic blood pressure [84,85]. This autoregulation property is impaired following traumatic brain injury, as shown in a rat model of severe drop-impact acceleration brain injury [86]. Most important, the loss of autoregulation in cerebral arteries in this scenario is rescued by mitochondria-targeted antioxidant mitoTEMPO and by scavenging of H_2_O_2_ [86]. It is noteworthy that cerebral artery mitochondria, when compared to other vascular beds, may be particularly sensitive to environmental insults. Indeed, rats subjected to microgravity via hind limb unweighting exhibit increased mitochondrial ROS, mitochondria PTP opening and malondialdehyde level, mitochondrial respiratory rate, membrane potential, and manganese superoxide dismutase (MnSOD)/glutathione peroxidase (GPx) activity ratio in cerebral but not mesenteric arteries [64]. Conceivably, treatment with nicotinamide adenine dinucleotide phosphate (NADPH) oxidase inhibitor apocynin and with mitochondria-targeted antioxidant mitoTempol favored recovery of cerebral arteries from oxidative stress, while these agents had no effect on mesenteric vessels [64].

## 5. Mitochondria during Fetal Development

Data on mitochondria during development are scarce. In chick embryos, developmental increase in cytochrome oxidase (complex IV) and citrate synthase (Krebs cycle enzyme) activity is reported [87]. Yet, this increase in activity is accompanied by a decrease in cytochrome oxidase subunit III mRNA and mitochondrial DNA levels across development [87]. In the ovine carotid artery that provides blood supply to the brain, metabolomics analysis also points at the mitochondria developmental changes [88]. 

Both ADH and two isozymes of ALDH (ALDH-I and ALDH-II-NAD+) are detected in rat liver samples as early as 5 days before birth [89]. This time window corresponds to the end of the second trimester in humans [90], which is close to the time-window for the detection of ALDH6A1 expression and functional activity in fetal baboon cerebral arteries [27]. Moreover, in the perinatal period, a remarkable increase in rat fetal liver ALDH activity is noted in the mitochondrial fraction [91]. 

## 6. Alcohol Modifications in Fetal Mitochondria Morphology and Function

Acute and chronic alcohol exposure during prenatal development results in impaired mitochondrial morphology and function as widely studied in various cell types and organs [92]. Abnormal mitochondrial morphology such as elongation, cristae disorientation, and appearance of a dense material in the matrix are described in the mitochondria of half-term mini-pig fetuses who were subjected to daily exposure to alcohol in utero (3g/kg of maternal body weight) [93]. Abnormal mitochondrial aggregation is noted in preputial neural tissues in newborns of alcohol-abusing mothers [94].

At the molecular level, alcohol-driven alterations in fetal mitochondrion have been linked to a variety of signaling pathways. *Acute* ethanol (2.5 mg/mL for 24 h) exposure of cultured fetal rat hepatocytes reduces mitochondrial complex I, complex IV, succinate dehydrogenase, and ADP translocase activities. These decreases are accompanied by a decrease in mitochondrial GSH level [92]. A similar ethanol exposure paradigm in cultured fetal rat cortical neurons leads to a rapid onset of oxidative stress that precedes cellular apoptosis [95]. Mitochondria-linked cellular apoptosis is also reported in an animal model of prenatal acute exposure to alcohol. In particular, gastric delivery of 4 g/kg ethanol to Sprague–Dawley rats on days 17, 18, and 19 of gestation leads to an increased mitochondrial permeability, release of cytochrome c and apoptosis-inducing factor from mitochondria, and increased level of lipid peroxidation product 4-hydroxynonenal in fetal whole-brain mitochondrion fraction [96].

Several studies ranging from cell cultures to animal models also report changes in mitochondrial function upon *protracted or chronic* alcohol exposure. For instance, rat primary cerebellar neuron cultures treated with 50 mM ethanol for 96 h have significantly reduced mRNA levels of mitochondrial genes encoding several electron transport chain complexes [97]. A four-day-long treatment of immature human PNET2 neuronal cultured cells with 100 mM ethanol decreases mitochondrial mass as detected by reduced mitochondrial protein expression and decreased fluorescence labeling with green mitochondrial dye MitoTracker [98]. Alterations in mitochondrion content are paralleled by a decreased mitochondrial function [98]. Deleterious effects of alcohol exposure in this setting are diminished by the broad-spectrum caspase inhibitors and are fully reversed by nerve growth factor stimulation [98]. 

At the organismal level, exposure of chick embryos to ethanol (75mg/100g of weight) on embryonic days 11, 13, 15, and 17 decreases cytochrome oxidase activity without alteration of cytochrome oxidase subunit III mRNA level [87]. In a mouse model, extended exposure to alcohol (gestational days 6 through 15) results in an increased fraction of immature mitochondria in fetal brain on gestational day 18 [99]. Reduced activities of respiratory chain complexes I and IV, as well as ATP synthase are also found [99]. 

Prenatal chronic alcohol exposure in the form of liquid diet from day 8 to delivery in mouse model results in depressed mitochondrial respiration and activities of the inner membrane enzymes cytochrome c oxidase and succinate dehydrogenase [29]. Oral chronic daily administration of ethanol (4 g/kg of weight) to timed pregnant guinea-pigs results in decreased mitochondrial level of GSH in the hippocampus of newborn progeny without change in cytosolic GSH concentration [100]. Thus, mitochondria may be particularly vulnerable to effects of alcohol. 

Moreover, when compared to adults, fetal mitochondria may be an overly sensitive target for alcohol. Indeed, prenatal alcohol exposure by five oral feedings of pregnant Sprague–Dawley dams with ethanol (4 g/kg of weigh, at 12 h intervals) on gestational days 17 through 19 results in an increased HNE level in fetal hepatocyte mitochondria when compared to their maternal counterparts [101]. This increase in fetal HNE level is arising from the higher susceptibility to HNE production and the lack of metabolic capacity [101]. On the other hand, intrauterine ischemia induced by a 30 min-long occlusion of the uterine artery results in decreased mitochondrial respiration in term (20 days of gestation) but not preterm (14 days of gestation) Wistar rat fetuses [102]. Although it is uncertain whether in utero ischemia may mimic alcohol exposure, the existence of specific time-periods that may constitute “windows of vulnerability” for fetal mitochondrial damage by environmental insult (including alcohol exposure) cannot be ruled out.

Persistency is another characteristic of mitochondrial changes in response to alcohol exposure during fetal period. Indeed, depressed mitochondrial function is observed in the early postnatal period in liver and brain tissues, including cerebellar neurons of rat pups that were exposed to alcohol prenatally [103,104]. Notably, while liver and brain mitochondria exhibit reduced ATP synthase activity, only the liver is characterized by decreased complex III activity [103]. In addition, none of the mitochondrial enzymes under study are affected in the heart [103]. Thus, fetal mitochondria vulnerability to alcohol is also tissue-specific. 

## 7. Alcohol and Fetal Cerebrovascular Mitochondria

Data on the physiological role of mitochondria and pathophysiological consequences of alcohol targeting of mitochondria in fetal cerebral artery are scarce. A recent study from our group utilized proteomics analysis to determine organelles and pathways that constitute major targets of prenatal alcohol exposure in fetal cerebral arteries. For this purpose, we focused on nonhuman primates, which offer several advantages to work on FASDs-related questions. These advantages include the large size of fetal cerebral arteries and intrauterine development that closely matches the developmental trajectory of human fetuses [20]. In our experimental paradigm, pregnant baboon (*Papio spp*) dams undergo three episodes of gastric alcohol infusions (1.8 g/kg ethanol, total infusion volume 200 mL) at gestational days 90, 100, and 110 [105,106,107]. The amount of alcohol in the infusion liquid results in 80 mg/dL of BAL in maternal blood samples and approximately 63 mg/dL alcohol in amniotic fluid [105]. With the average gestation in baboons lasting 163–185 days [108], the chosen interval for alcohol infusions corresponds to mid-pregnancy in humans. The control group of pregnant baboons receives three gastric infusions of orange-flavored drink that matches caloric count of alcohol-containing infusion liquid. Fetuses are delivered by cesarean section near-term at 165 days of gestation, and fetal basilar (cerebral) arteries are harvested for differential protein expression (proteomics) analysis [27]. Of note, the basilar artery participates in irrigation of the cerebellum that constitutes a rather sensitive target for prenatal alcohol exposure. In an ovine model of pregnancy, fetal alcohol exposure results in selective increase of cerebral blood flow in cerebellum, with subsequently detected neuronal loss in this region [109].

**Figure 3:**
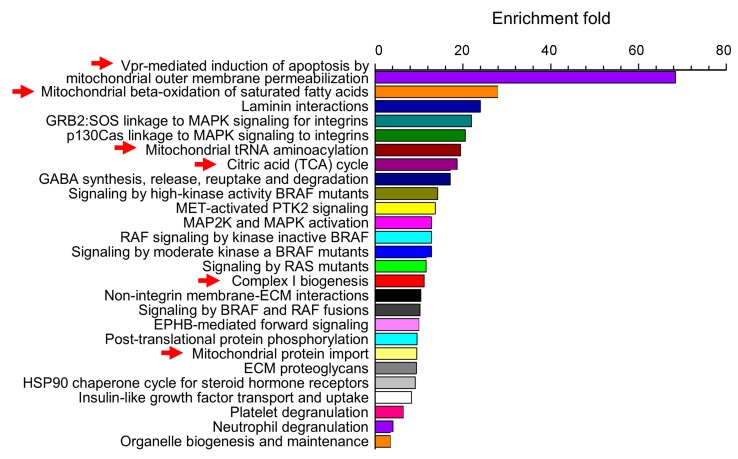
Statistical enrichment analysis points to the statistically significant overrepresentation of mitochondria-related pathways within the set of baboon fetal cerebral artery proteome that is sensitive to prenatal alcohol exposure. Enrichment fold refers to the increase in appearance of proteins within each pathway when compared to the relative distribution of these proteins within *Homo sapience* proteome. Thus, proteins on this plot do not reflect frequency of appearance in the overall proteome but rather a group of proteins that is selectively targeted by prenatal alcohol exposure. With modifications from [27].

Differential protein expression analysis based on mass spectroscopy identification reveals statistically significant differences in the relative abundance of 238 proteins between control and alcohol-exposed fetal basilar arteries [27]. Statistical enrichment analysis points to the statistically significant overrepresentation of mitochondria-related proteins within this set. These proteins represent Vpr-mediated induction of apoptosis by mitochondrial outer membrane permeabilization, fatty acid beta-oxidation, tRNA aminoacylation, complex I biogenesis pathways, as well as Krebs cycle (Figure 3).

These findings from our group point to the mitochondrial proteome as one of the components within fetal cerebral arteries that is highly sensitive to alcohol exposure. Our conclusion on mitochondrial sensitivity to prenatal alcohol exposure is consistent with previously published observations on significant alterations in mitochondrial transcriptome and proteome in liver mitochondria from ethanol-fed rats [110,111]. Importantly, in our experimental paradigm, baboon fetuses were subjected to only three alcohol exposure episodes with moderate alcohol levels [27,105]. Yet, such exposure renders *persistent* changes in mitochondrial proteome, as there is a month-long period of alcohol-free gestation between last alcohol exposure (110 days of gestation) and fetal tissue harvesting near-term (165 days of gestation) [27].

In our baboon model, it remains to be determined whether mitochondrion functionality is affected by alcohol. Consistent with proteomics analysis, we documented increases in protein level on Western blot and increased activity of ALDH6A1 enzyme in fetal baboon cerebral artery lysates from alcohol-exposed fetuses when compared to control [27]. However, a large-scale functional study on fetal cerebrovascular mitochondria function following prenatal alcohol exposure remains to be conducted. If mitochondrial function is unchanged, then observed changes in mitochondrial proteome may be indicative of the compensatory mechanisms that develop near-term in response to a depression of mitochondrial activities by preceding exposure to alcohol. The presence of such compensatory mechanisms would be consistent with previous findings in fetal mini-pigs: While hepatocyte mitochondrion from *half-term* mini-pig fetuses following prenatal alcohol exposure shows abnormal morphology, alterations in mitochondrial morphology and function are absent in *term-fetuses* subjected to a similar alcohol exposure paradigm [112]. 

Although acute alcohol application dilates cerebral arteries from baboon mid-pregnancy fetuses in vivo and in vitro [105,106,107], basic cerebrovascular properties of the *near-term* fetal baboons that are subjected to prenatal alcohol exposure remain similar to control groups when evaluated both in vivo and in vitro [106,107]. While we cannot evaluate every possible aspect of arterial basic function, it is plausible to assume that fetal cerebral artery recovers or develops compensatory mechanisms in response to alcohol exposure in utero. In this regard, higher susceptibility of cerebral artery mitochondria to insult later in life cannot be ruled out. Indeed, prenatal alcohol exposure in a C57BL6 mouse model (3 g/kg of weigh ethanol on gestational days 12.5 through 15.5) results in apparently normal basic function of cerebral arteries yet manifests in a worse neurological recovery outcome after transient cerebral ischemia in adult 3-months-old progeny [113]. Increased susceptibility of the brain to damage following ischemia/reperfusion in adulthood is also reported in Sprague–Dawley rats fed 3% ethanol liquid diet for the duration of their pregnancy [114]. Considering that mitochondrial function plays a critical role in cellular recovery from ischemia/reperfusion [102,115,116,117], the role of mitochondria capacity to cope with the environmental stress following prenatal alcohol exposure should be explored systematically.

## 8. Conclusions

Current literature points to mitochondria as a potential link between prenatal alcohol exposure and the brain damage that is characteristic of FASDs. In this regard, cerebrovascular mitochondria hold several unanswered questions. First, there is lack of studies that focus on possible changes in *cerebrovascular* mitochondria morphology and function following prenatal alcohol exposure. What is the “window of vulnerability” for this cellular organelle in cerebrovascular tissue? Are changes in cerebral artery proteome and function driven by prenatal alcohol exposure and/or damage by alcohol metabolites, such as acetaldehyde? Is the alcohol effect on cerebral artery mitochondria direct or mediated by an alcohol-/metabolite-driven changes in fetal cerebral artery diameter and, thus, oxygenation? These questions must be answered in order to adapt biomedical research findings to therapeutic needs. 

Moreover, the question of applicability of findings in animal models to human populations remains open. Even findings in primate animal models should be interpreted with caution. Overall, human developmental expression trajectories are more similar to monkey than rodent [118]. However, comparison of brain transcriptome between humans and macaque points at ontogeny and phylogeny-specific points of divergence [119]. Thus, validation of primate findings on humans is still required. 

Despite many open questions and apparent limitations in our current understanding of the role that fetal cerebral artery mitochondria play in the deleterious sequelae of FASDs, mitochondria research still holds high promise. Mitochondria-targeted interventions with ROS scavengers and antioxidants are being increasingly considered as a preventive and therapeutic measure against alcohol-driven mitochondria damage in various organs [61]. Moreover, reversal and prophylaxis of mitochondrial damage by aberrations in intrauterine blood supply is being consistently studied, with ascorbic acid, alpha-tocopherol, and melatonin showing promise in reducing fetal brain damage [116,120]. Considering that alcohol-driven changes in fetal cerebrovascular mitochondria could arise from both direct targeting by alcohol [27,61] and secondary damage via alcohol-driven alterations in fetal cerebral blood flow [105,106], fetal cerebrovascular mitochondria likely constitute a promising target for anti-FAS/FASDs therapeutic intervention.

## Abbreviations

ADH	alcohol dehydrogenase
ALDH	aldehyde dehydrogenase
AngII	angiotensin II
ATP	adenosine triphosphate
BAL	blood alcohol level
BK_Ca_	voltage- and Ca^2+^-gated potassium channels of large conductance
EC	endothelial cell
FAS	fetal alcohol syndrome
FASDs	fetal alcohol spectrum disorders
GPx	glutathione peroxidase
HNE	4-hydroxynonenal
MCA	middle cerebral artery
MCU	mitochondrial Ca^2+^ uniporter
MnSOD	manganese superoxide dismutase
PTP	permeability transition pore
NAD^+^	nicotinamide adenine dinucleotide
NADPH	nicotinamide adenine dinucleotide phosphate
NOS	nitric oxide synthase
SR	sarcoplasmic reticulum
PI3K	phophoinositide-3 kinase
ROS	reactive oxygen species
RyR	ryanodine receptor
VSM	vascular smooth muscle
